# Association between N-terminal pro-brain natriuretic peptide levels and outcomes of ischemic stroke: A systematic review and meta-analysis

**DOI:** 10.1371/journal.pone.0322816

**Published:** 2025-06-27

**Authors:** Yehong Zhang, Jingjia Zheng, Fangyong Xu

**Affiliations:** Emergency Department, Changxing County People’s Hospital, Changxing County, Zhejiang Province, China; The University of Tokyo Graduate School of Medicine Faculty of Medicine, JAPAN

## Abstract

**Background:**

N-terminal pro-brain natriuretic peptide (NT-proBNP) was identified as an important biomarker of cardiovascular disease, in ischemic stroke. This study intends to assess the association of NT-proBNP levels with clinical outcomes of patients ischemic stroke patients.

**Methods:**

A comprehensive search of MEDLINE, Web of Science, ScienceDirect, and Cochrane CENTRAL electronic databases was done for papers published till April 2024 and reporting on the levels of NT-proBNP in patients with ischemic stroke. Outcomes of interest included mortality (all-cause and cardiovascular) and neurological, and functional outcomes. A random-effects meta-analysis model was used, and final estimates were reported as pooled odds ratio (OR) with 95% confidence interval (CI).

**Results:**

Elevated NT-proBNP levels were significantly linked to increased all-cause (pooled OR = 2.322, 95% CI: 1.718 to 2.925) and cardiovascular mortality (pooled OR = 1.797, 95% CI: 1.161 to 2.433). Higher NT-proBNP levels were also related to poorer functional outcomes (pooled OR = 1.129, 95% CI: 1.041 to 1.217). Patients with higher NT-proBNP levels had somewhat worse neurological outcomes (pooled OR = 1.317, 95% CI: 0.859 to 1.774). Considerable heterogeneity was detected across the studies (I² > 40% in most analyses).

**Conclusion:**

NT-proBNP levels may serve as a robust predictor of mortality and offer potential utility in predicting functional recovery in ischemic stroke patients. The integration of NT-proBNP measurement into clinical settings may be beneficial for risk stratification and management of stroke survivors.

## Introduction

Ischemic stroke is considered one of the major contributors to mortality and long-term disability worldwide [1]. Therefore, identification of biomarkers that can predict outcomes and guide therapeutic strategies in stroke patients is crucial [2].

N-terminal pro-brain natriuretic peptide (NT-proBNP) has been identified as a potential biomarker of considerable interest due to its function and association with cardiovascular diseases [3].

NT-proBNP is a prohormone produced primarily in the cardiac ventricles [4]. It is released in response to changes in heart wall stress, serving as a regulatory peptide that modulates blood pressure and fluid balance. In clinical practice, NT-proBNP is predominantly recognized as a marker for diagnosing and managing heart failure [4], since elevated levels of this peptide indicate an increased strain on the cardiac walls, which is commonly seen in conditions such as heart failure, acute coronary syndromes, and atrial fibrillation [4].

The connection between cardiovascular impairment and stroke also makes NT-proBNP a promising candidate marker of the risk of ischemic stroke [5]. Ischemic stroke occurs as a result of obstruction (due to a thrombus or embolism) that compromises blood supply to parts of the brain, resulting in neuronal injury [6]. In addition to local cerebral changes, this pathological state also leads to systemic physiological responses, including changes in cardiac function. Therefore, current research suggests that measuring NT-proBNP levels post-stroke may reflect not just concurrent cardiac pathology but also severity of stroke and the extent of neurohormonal activation triggered by the acute event [7].

Several observational studies have reported associations between elevated NT-proBNP levels and poor outcomes in stroke patients, such as higher mortality rates, increased risk of complications, and diminished functional recovery [8–11]. These outcomes may be mediated through NT-proBNP’s relationship with left ventricular dysfunction, atrial fibrillation, and the extent of brain damage, all of which are critical determinants of prognosis after stroke. Furthermore, as shown by recent research, neuroendocrine stress caused by acute stroke may lead to increased NT-proBNP levels. NT-proBNP levels, therefore, may serve as a measure of the intensity of physiological stress response [12].

However, despite accumulating evidence, the prognostic value of NT-proBNP in the management of stroke patients remains unclear, potentially due to high that stems from differences in study design, timing of biomarker measurement, patient demographics, and the presence of concomitant cardiac conditions.

This review investigated the association between NT-proBNP and the outcomes of ischemic stroke to assess the prognostic value of NT-proBNP in this group of patients.

## Methods

### Eligibility criteria

Experimental trials and/or case-control, cohort, and cross-sectional observational studies that investigate the relationship between the levels of NT-proBNP and outcomes of ischemic stroke were eligible for inclusion. Full-text articles were included, while publications that were available solely as abstracts or unpublished data were excluded. Only studies that compared the effects of varying levels of NT-proBNP in patients undergoing treatment for ischemic stroke were eligible. The included studies were required to meet the following criteria:

### Study population

Adults diagnosed with ischemic stroke, that had measurements of NT-proBNP taken during their treatment course.

### Exposure

Observational data on NT-proBNP levels, without specific interventions that may alter these levels.

### Outcomes of interest

#### All-cause mortality.

Total number of deaths from any cause during the study follow-up period.

#### Cardiovascular mortality.

Number of deaths specifically attributed to cardiovascular causes post-stroke.

#### Functional status.

Assessment of patients’ functional independence post-stroke, potentially measured by scales such as the Barthel Index or the modified Rankin Scale.

#### Neurological outcome.

Evaluation of neurological impairment.

#### Haemorrhagic transformation.

Incidence of haemorrhagic transformation in stroke, identified either through clinical assessment or radiological imaging.

### Search strategy

Literature search was performed across MEDLINE, Web of Science, ScienceDirect, and Cochrane CENTRAL databases from the inception to 30 April 2024, with no language restrictions. Search terms and combinations used were: “N-terminal pro-brain natriuretic peptide,” “NT-proBNP,” “ischemic stroke,” “stroke outcomes,” “biomarkers in stroke,” and “neurological assessment.” Manual searches of bibliographies from selected studies were conducted to identify additional relevant articles.

### Study registration

The protocol of this systematic review was registered at PROSPERO, with the number: CRD42024547558. Only publicly available studies were utilized for this meta-analysis and no ethical approval was required.

### Study selection procedure

Two independent reviewers performed study selection. Initially, each reviewer conducted a separate search and then collaboratively reviewed titles and abstracts to shortlist preliminary eligible articles. Subsequently, full texts of selected studies were independently assessed by each reviewer to confirm eligibility based on the established criteria. In cases of disagreement, discussion between the authors or consultation with additional reviewer were used to reach consensus. List of excluded studies with reasons are attached as S1 File.

### Data collection

A structured data collection framework was implemented under the guidance of the lead researcher. Retrieved data included date of data extraction, study title, author information, study design details, descriptions of the study population and setting, sample size for each group, baseline participant characteristics, and outcomes assessed. Additionally, information on the inclusion and exclusion criteria, descriptions of the groups based on NT-proBNP levels (high, normal, or not measured), follow-up duration, and key outcomes such as all-cause mortality, cardiovascular mortality, functional status, neurological outcomes, and incidences of haemorrhagic transformation were also extracted and catalogued.

### Risk of bias assessment

Study quality was evaluated by two independent reviewers using the Newcastle-Ottawa Scale (NOS) that assesses quality of non-randomized, observational studies [13]. The NOS provides a framework for evaluating potential bias in three critical areas: selection of participants, comparability of study groups, and the accuracy of the outcome or exposure measurement reported. Studies scoring 7–9 were deemed to have a ‘low’ risk of bias, those scoring 4–6 as having a ‘moderate’ risk of bias, and studies with scores from 0 to 3 had a ‘high’ risk of bias.

### Statistical analysis

A random-effects model through the inverse variance method of analysis was used [14] to accommodate differences among the included studies. For each study, odds ratios (ORs) with 95% confidence intervals (CIs) were calculated for both adjusted and unadjusted results. The pooled estimates were visually represented using Forest plots.

Heterogeneity among the studies was measured by chi-square tests and the I^2^ statistic. A chi-square test p-value below 0.05 and an I^2^ statistic exceeding 50% were used as indicators of significant heterogeneity [14]. Additional subgroup analysis and meta-regression was performed to identify the source of heterogeneity.

Funnel plots and Egger’s test were used to assess potential publication bias [14]. STATA software, version 16 was used for analyses. The dataset is available as S2 File.

## Results

### Search results

**Fig 1:**
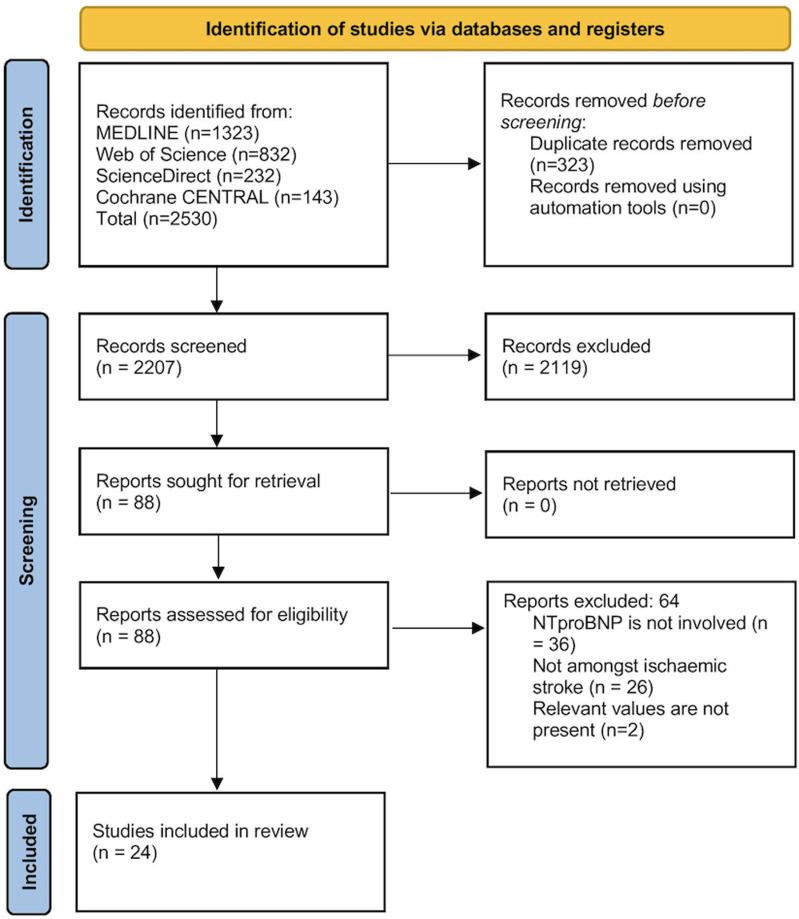
PRISMA flowchart.

Systematic literature search of the databases identified 4,173 articles. Of them, 2,851 records remained after deduplication. After thorough assessment for eligibility, 2,119 records were excluded primarily due to not reporting data on NT-proBNP, not focusing on ischemic stroke, or due to the lack of relevant outcome data. Finally, 24 studies were included in the review (Fig 1) [8–11,15–34].

### Characteristics of the included studies

Most included studies were prospective, and conducted across various countries including China, Austria, England, Slovenia, Denmark, Korea, Spain, Thailand, UK, and the USA (Table 1). Sample sizes ranged notably, from 67 to 6,315 participants. Most studies reported mean age of participants, often in the late sixties to early seventies. The studies explored outcomes such as mortality, functional status, and haemorrhagic transformation post-stroke, with follow-up periods varying from the duration of hospitalization to up to 14 months. In most studies, functional outcomes were assessed as measures of post-stroke disability or independence. Commonly used instruments included the modified Rankin Scale (mRS) and the Barthel Index. For example, several studies dichotomized functional outcome using mRS (with cutoffs such as mRS ≤ 2 or ≤3 to indicate favourable recovery), while others used alternative scales or categorization methods. Despite these minor differences, the underlying construct—namely, the degree of functional independence or disability—remained consistent across studies.

**Table 1:** Characteristics of the included studies.

Author and year of publication	Study Country	Study design	Participant details	Blood collection	Diagnosis method	Outcome	Cut-off	Follow up duration	Sample size	Mean age	Gender distribution (M/F)
Chang 2014	China	Prospective	Patients with AIS	<4 hrs	ECLIA	Functional outcome	>1500 pg/ml	3 months	217	61 (54-74)	150/67
Chen 2012	China	Prospective	Patients who were within three days of onset of acute ischemic stroke	<72 hrs	ELISA	Mortality	>1583.5 pg/ml	During hospitalization	122	71.5 ± 9.8	60/39
Dieplinger 2017	Austria	Prospective	Patients with AIS	<24 hrs	Chemiluminescent microparticle immunoassay	Mortality	>447 pg/mL	3 months	721	76 (66–84)	374/347
Greisenegger 2015	England	Prospective	People with Transient Ischemic Attack and Minor Ischemic Stroke (an acute loss of focal brain or monocular function with symptoms lasting >24 hours)	Admission	ELISA	Mortality, Vascular mortality	NR	76 months	929	74 (64–83)	460/469
Hajdinjak 2012	Slovenia	Prospective	Patients with acute stroke	Admission	ECLIA	Mortality	NR	During hospitalization	106	NR	62/44
Hatab 2022	Austria	Prospective	Patients with AIS	<24 hrs	ECLIA	Mortality	>794pg/mL	3 months	405	70.3 ± 13.4	233/172
Jenson 2012	Denmark	Prospective	Patients with AIS	<4 hrs	ECLIA	Mortality, Vascular mortality	NR	52 months	193	NR	109/84
Jenson 2006	Denmark	Prospective	Patients with AIS	<4 hrs	ECLIA	Mortality	>615 pg/mL	6 months	250	69 (51–85)	133/117
Lee 2015	Korea	Prospective	Patients with acute ischemic stroke or transient ischemic attack with a lesion	<24 hrs	ECLIA	Mortality, functional outcome	NR	6 months	410	67.7 ± 14.2	212/198
Liu 2020	China	Prospective	Patients aged ≥ 22 years who were diagnosed with ischemic stroke confirmed by computed tomography or magnetic resonance imaging of the brain within 48 h of symptom onset	<24 hrs	ELISA	Mortality, CVD	NR	12 months	2694	62.4 ± 10. 8	1714/980
Ma 2024	China	Retrospective	Patients with acute cerebral infarction treated with intravenous thrombolysis,	Admission	NR	Hemorrhagic transformation	NR	During hospitalization	197	68.10 ± 12.21	134/63
Rost 2011	UK	Prospective	Patients aged >18 years admitted to our stroke unit	<24 hrs	ELISA	Mortality, functional outcome	NR	6 months	569	67.9 ± 15	307/262
Ruiz-Franco 2023	Spain	Retrospective	Patients with AIS	<48 hrs	ECLIA	Neurological outcome	>326 pg/mL	During hospitalization	476	NR	214/262
Shen 2021	China	Retrospective	Hospitalized patients with AIS	Admission	NR	Functional outcome	NR	3 months	403	67 (56–75)	256/147
Srisujikul 2023	Thailand	Prospective	People were 18 or older and had a stroke onset	<72 hrs	ELISA	Functional outcome	>476 pg/mL	3 months	67	66.9 (13.9)	29/38
Tu 2013	China	Prospective	Patients with AIS	<24 hrs	CLIA	Mortality, functional outcome	>450 pg/mL	3 months	189	66	117/72
Tu 2017	China	Prospective	Patients had a first-ever AIS defined on admission	<48 hrs	ECLIA	Mortality, cardiovascular mortality	NR	12 months	6315	NR	NR
Wang 2020	China	Prospective	Patients with AIS	<24 hrs	ELISA	Functional outcome	NR	14 days	3216	6 2.5 ± 10.8	2048/1168
Whiteley 2012	England	Prospective	Patients with AIS	<24 hrs	ECLIA	Functional outcome	NR	3 months	270	NR	NR
Yang 2017	China	Prospective	Patients with AIS	<24 hrs	ELISA	Mortality, functional outcomes	NR	12 months	3126	62.5 ± 10.8	2048/1078
Zhang 2021	China	Retrospective	Patients with ischemic stroke who underwent intravenous thrombolysis	<24 hrs	NR	Mortality, functional outcome, Hemorrhagic transformation, Early neurologic deterioration	NR	3 months	447	NR	NR
Zhang X 2021	China	Retrospective	Patients with AIS	<24 hrs	Electro chemiluminescence immunoassay	Mortality	>1113.5 pg/mL	3 months	1039	70 ± 13	635/404
Zhao 2020	China	Retrospective	Patients with AIS	During the first week after onset	NR	Mortality, functional outcome	>431 pg/mL	During hospitalization	550	71 ± 9	314/236
Zhu 2023	China	Retrospective	AIS patients who had rt-PA intravenous thrombolysis	NR	NR	END (early neurological deterioration)	NR	90 days	325	68 (59–76)	208/117

In contrast, neurological outcomes were generally defined as the evaluation of neurological impairment post-stroke. Although many studies relied on established clinical scales (such as the National Institutes of Health Stroke Scale, NIHSS), the specific thresholds or time points for assessment varied. For instance, one study might have defined neurological outcome in terms of early neurological deterioration (END) during the acute phase, whereas another assessed neurological status at a later follow-up. This heterogeneity in assessment tools and timing reflects the absence of a universally adopted definition for neurological outcomes in the context of stroke studies. As identified by the NOS score, 11 studies had a high risk of bias, 9 had a moderate risk, and 4 studies had a high risk of bias (Table 2).

**Table 2:** Risk of bias assessment of the included studies.

Author and year of publication	Selection	Comparability	Outcome	Risk of bias (Total score)
Chang 2014	2 points	2 points	3 points	Low (7)
Chen 2012	2 points	2 points	1 point	Moderate (5)
Dieplinger 2017	3 points	2 points	3 points	Low (8)
Greisenegger 2015	2 points	2 points	3 points	Low (7)
Hajdinjak 2012	2 points	2 points	1 point	Moderate (5)
Hatab 2022	3 points	2 points	3 points	Low (8)
Jenson 2012	2 points	2 points	3 points	Low (7)
Jenson 2006	1 point	1 point	1 point	High (3)
Lee 2015	3 points	2 points	2 points	Low (7)
Liu 2020	2 points	2 points	3 points	Low (7)
Ma 2024	1 point	1 point	0 points	High (2)
Rost 2011	3 points	2 points	3 points	Low (8)
Ruiz-Franco 2023	1 point	1 point	1 point	High (3)
Shen 2021	2 points	2 points	1 point	Moderate (5)
Srisujikul 2023	3 points	2 points	2 points	Low (7)
Tu 2013	2 points	2 points	3 points	Low (7)
Tu 2017	3 points	2 points	3 points	Low (8)
Wang 2020	2 points	2 points	2 points	Moderate (5)
Whiteley 2012	1 point	2 points	3 points	Moderate (6)
Yang 2017	2 points	2 points	1 point	Moderate (5)
Zhang 2021	1 point	1 point	1 point	High (3)
Zhang X 2021	2 points	2 points	2 points	Moderate (6)
Zhao 2020	2 points	2 points	1 point	Moderate (5)
Zhu 2023	2 points	2 points	2 points	Moderate (6)

### Mortality

**Fig 2:**
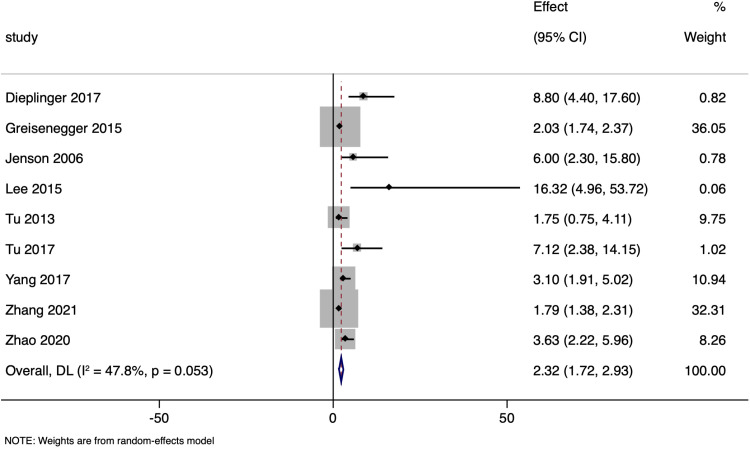
Forest plot showing the association between N-terminal pro brain natriuretic peptide levels and all-cause mortality amongst ischaemic stroke patients (unadjusted estimates).

Nine studies assessed the link between NT-proBNP levels and mortality in ischemic stroke patients, using unadjusted odds ratios. The pooled OR of 2.322 (95% CI: 1.718 to 2.925) pointed to a significant association (p-value < 0.001), with moderate heterogeneity (I² of 47.8%; Fig 2).

**Fig 3:**
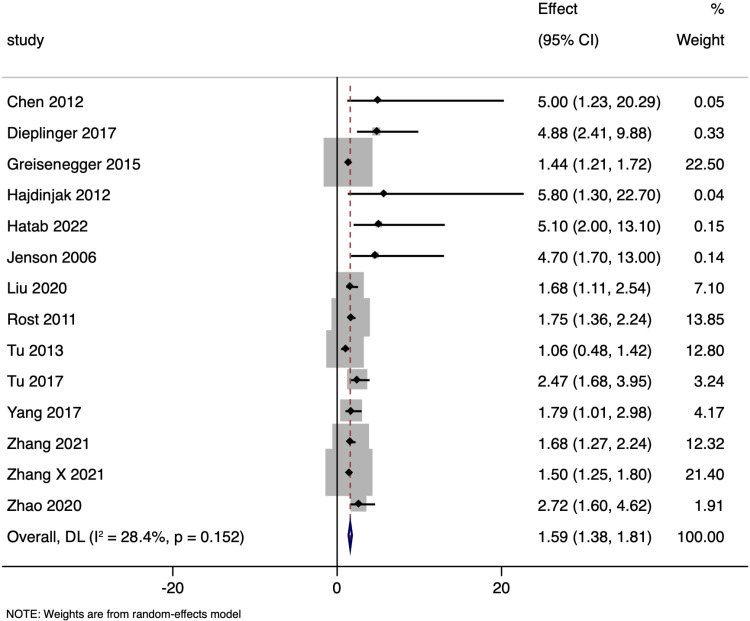
Forest plot showing the association between N-terminal pro brain natriuretic peptide levels and all-cause mortality amongst ischaemic stroke patients (adjusted estimates).

Fourteen studies reported the link between adjusted NT-proBNP levels and mortality in ischemic stroke patients using adjusted odds ratios. The analysis revealed a pooled adjusted OR of 1.591 (95% CI: 1.376 to 1.806), demonstrating a statistically significant association (p < 0.001) (Fig 3), with low (I² = 28.4%) heterogeneity. Funnel plot was asymmetrical (S1 Fig), which was confirmed by significant Egger’s test (p = 0.001). A trim-and-fill analysis was conducted to address potential publication bias. The original random-effects model based on 14 studies estimated a pooled effect of 1.591 (95% CI: 1.376–1.806). After imputing 6 missing studies (for a total of 20 studies), the adjusted pooled effect was 1.555 (95% CI: 1.315–1.795), indicating only a modest attenuation of the effect size due to publication bias.

**Fig 4:**
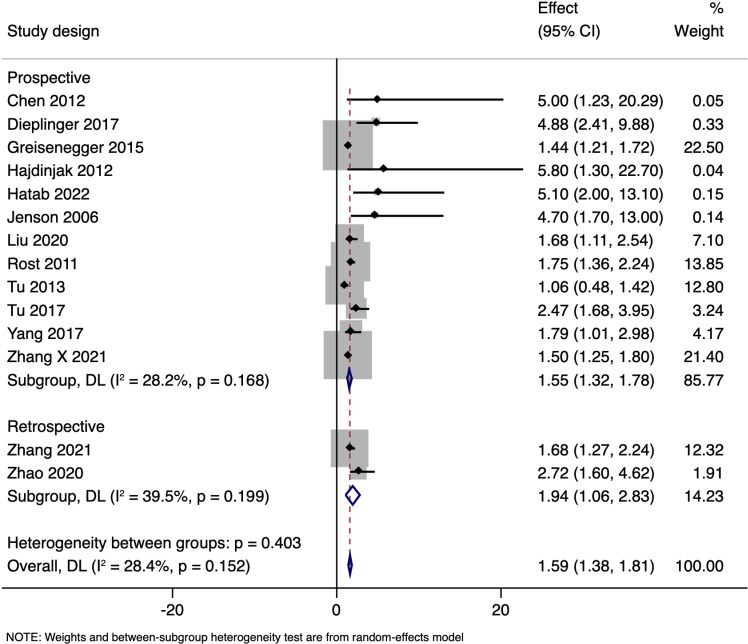
Subgroup analysis based on study design showing the association between N-terminal pro brain natriuretic peptide levels and all-cause mortality amongst ischaemic stroke patients.

Subgroup analysis using adjusted estimates stratified by study design showed that the results pooled from 12 prospective studies had adjusted OR of 1.553 (95%CI: 1.321 to 1.784), while two retrospective studies provided a combined adjusted OR of 1.944 (95% CI: 1.057 to 2.831). There was no difference in estimates between the subgroups (p = 0.403) (Fig 4).

**Fig 5:**
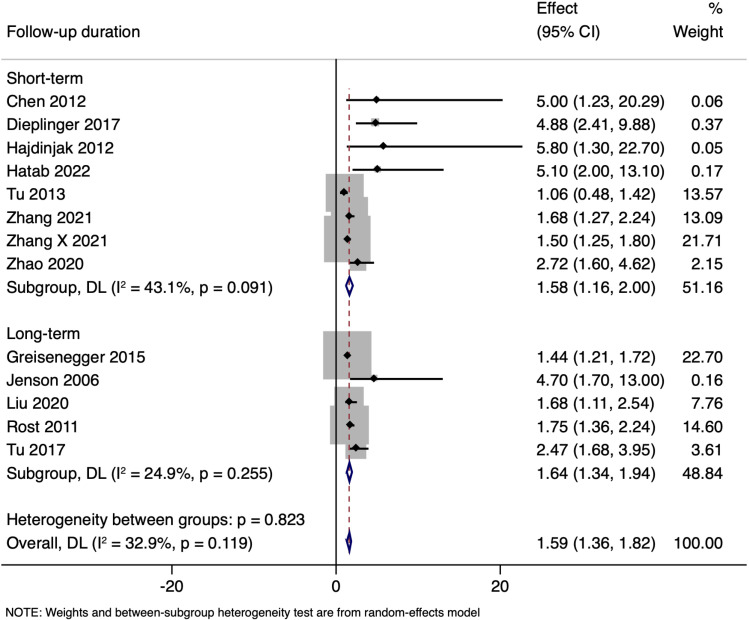
Subgroup analysis based on duration of follow-up showing the association between N-terminal pro brain natriuretic peptide levels and all-cause mortality amongst ischaemic stroke patients.

Subgroup analysis based on follow-up duration revealed that the results pooled from eight studies with short-term follow-up had an adjusted OR of 1.580 (95% CI: 1.163 to 1.997), while five studies with long-term follow-up provided a combined adjusted OR of 1.639 (95% CI: 1.341 to 1.936). Estimates of the short-term and long-term follow-up subgroups were comparable (p = 0.823) (Fig 5). Each subgroup analysis demonstrated significant effects (Short-term: p < 0.001; Long-term: p < 0.001), indicating that NT-proBNP levels consistently predict mortality across different follow-up durations (Table 3).

**Table 3:** Subgroup analysis results of the mortality and functional outcome.

Subgroup categories	Pooled OR (95%CI)	p-value (difference between subgroups)
**Mortality**		
**Study design**		
Prospective	1.553 (1.321 to 1.784)	0.403
Retrospective	1.944 (1.057 to 2.831)	
**Follow-up duration**		
Short-term (<6 months)	1.580 (1.163 to 1.997)	0.823
Long-term (6 months or more)	1.639 (1.341 to 1.936)	
**Timing of NT-proBNP measurement**		
Admission	1.442 (1.188 to 1.697)	0.918
<24 hours	1.559 (1.302 to 1.816)	
**Risk of bias findings**		
Low risk	1.594 (1.229 to 1.958)	0.920
Moderate risk	1.562 (1.301 to 1.823)	
High risk	1.824 (0.562 to 3.087)	
**Functional outcome**		
**Study design**		
Prospective	1.161 (0.976 to 1.346)	0.436
Retrospective	1.320 (0.964 to 1.676)	
**Risk of bias**		
Low risk	1.153 (0.791 to 1.514)	0.567
Moderate risk	1.240 (1.020 to 1.460)	
High risk	1.360 (1.160 to 1.590)	

We further conducted subgroup analyses to explore whether the timing of NT-proBNP measurement and study quality influenced the association with adjusted mortality. For the timing-based analysis, studies were categorized into two groups: “Admission” and “<24 hours.” In the “Admission” subgroup, pooled adjusted OR was 1.442 (95% CI: 1.188–1.697) with minimal heterogeneity (Cochran’s Q = 0.64, p = 0.425; I² = 0.0%) (S2 Fig). In contrast, the “<24 hours” subgroup—which included eight studies yielded a pooled adjusted OR of 1.559 (95% CI: 1.302–1.816) with moderate heterogeneity (Cochran’s Q = 10.33, p = 0.171; I² = 32.2%) (S3 Fig). Additionally, subgroup analysis by risk of bias stratified studies into low, moderate, and high risk groups. The low risk subgroup (n = 7 studies) demonstrated a pooled adjusted OR of 1.594 (95% CI: 1.229–1.958); the moderate risk subgroup (n = 5 studies) had a pooled adjusted OR of 1.562 (95% CI: 1.301–1.823); and the high risk subgroup (n = 2 studies) showed a pooled adjusted OR of 1.824 (95% CI: 0.562–3.087) (S4 Fig). Meta-regression was performed with all the variables used in subgroup analysis and NTproBNP cut-off value (as continuous variable). However, none of the variables were able to explain heterogeneity associated with this outcome with every univariable meta-regression model giving a p-value more than 0.05.

### Cardiovascular mortality

**Fig 6:**
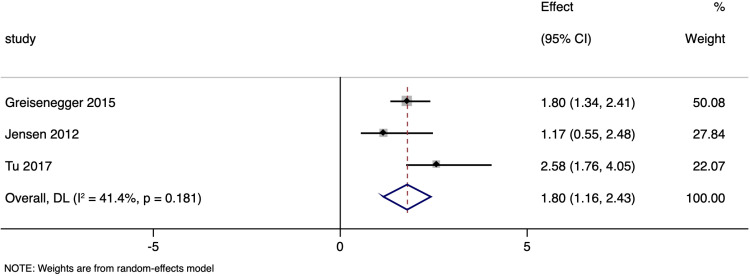
Forest plot showing the association between N-terminal pro brain natriuretic peptide levels and cardiovascular mortality amongst ischaemic stroke patients.

Three studies examined the link between NT-proBNP levels and cardiovascular mortality in ischemic stroke patients, with a pooled adjusted OR of 1.797 (95%CI: 1.161 to 2.433; p < 0.001) (Fig 6), with moderate (I² = 41.4%) heterogeneity. No subgroup analysis or publication bias assessment were done due to limited number of studies.

### Neurological outcome

**Fig 7:**
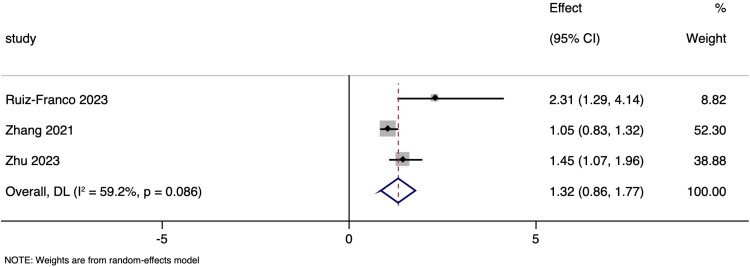
Forest plot showing the association between N-terminal pro brain natriuretic peptide levels and neurological outcomes amongst ischaemic stroke patients.

As shown in Fig 7, 3 studies that investigated the link between NT-proBNP levels and neurological outcomes post-stroke, reported insignificant (p > 0.05) pooled OR of 1.317 (95%CI: 0.859 to 1.774). This finding suggests that higher NT-proBNP levels may be associated with poorer neurological outcomes post-stroke. The heterogeneity was considerable, with an I² of 59.2%. Subgroup analysis and publication bias assessment were not done due to small number of studies.

### Haemorrhagic transformation

**Fig 8:**
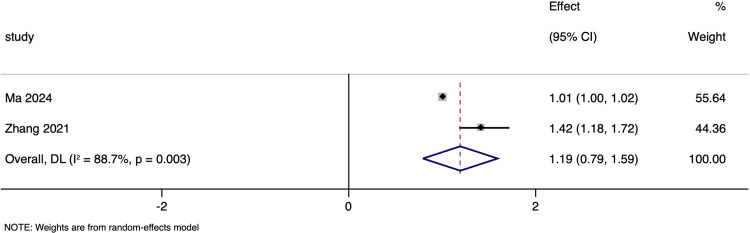
Forest plot showing the association between N-terminal pro brain natriuretic peptide levels and haemorrhagic transformation amongst ischaemic stroke patients.

Two studies investigated the association between NT-proBNP levels and haemorrhagic transformation, and reported pooled adjusted OR of 1.192 (95%CI: 0.793 to 1.591), which indicates no significant relationship (p > 0.05) (Fig 8), with substantial heterogeneity (I² of 88.7%).

### Functional outcome

**Fig 9:**
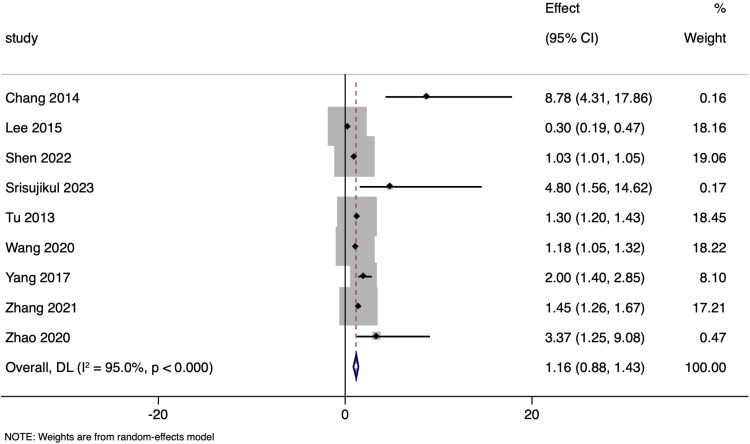
Forest plot showing the association between N-terminal pro brain natriuretic peptide levels and functional outcomes amongst ischaemic stroke patients (unadjusted estimates).

Nine studies explored the association between NT-proBNP levels and unadjusted functional outcomes following ischemic stroke. The pooled unadjusted OR was 1.155 (95%CI: 0.883 to 1.427) (Fig 9). However, the heterogeneity among the included studies was extremely high, with an I² of 95.0%

**Fig 10:**
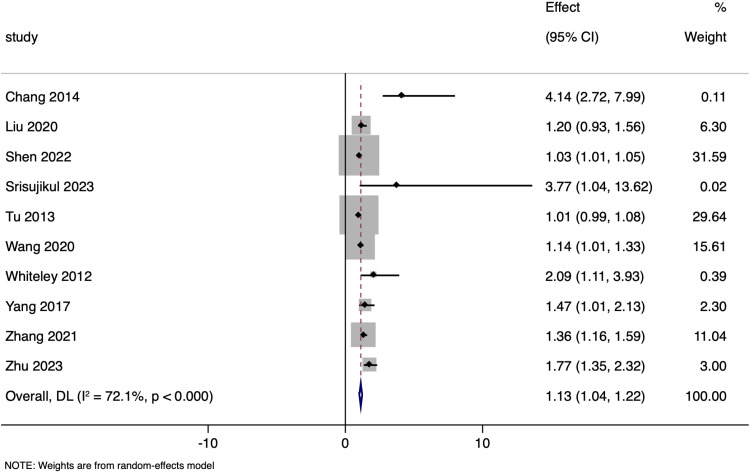
Forest plot showing the association between N-terminal pro brain natriuretic peptide levels and functional outcomes amongst ischaemic stroke patients (adjusted estimates).

Ten studies assessed the association between NT-proBNP levels and adjusted functional outcomes following ischemic stroke, with the pooled adjusted OR of 1.129 (95%CI: 1.041 to 1.217), a statistically significant effect (p < 0.001) (Fig 10). There was a considerable heterogeneity (I² = 72.1%), and Funnel plot was asymmetrical with significant Egger’s test (p = 0.001) (S5 Fig). The original random-effects meta-analysis based on 10 studies estimated a pooled effect of 1.129 (95% CI: 1.041–1.217). After imputing 5 potentially missing studies (increasing the total to 15 studies), the adjusted pooled effect was 1.098 (95% CI: 1.000–1.196). Although the effect size was modestly attenuated, the association remains statistically significant, suggesting that publication bias may slightly overestimate the effect but does not substantially alter the overall findings.

Subgroup analysis based on study design showed that seven prospective studies contributed to a pooled adjusted OR of 1.161 (95%CI: 0.976 to 1.346) (Table 3). Three retrospective studies showed a combined adjusted OR of 1.320 (95% CI: 0.964 to 1.676) (S6 Fig). The analysis highlighted no significant difference in effect sizes between the prospective and retrospective study designs (p = 0.436), suggesting that NT-proBNP’s predictive value for functional outcomes post-stroke is consistent across different study methodologies.

We performed a subgroup meta‐analysis including only studies where NT-proBNP was measured within 24 hours of stroke onset. This analysis, which included six studies, yielded a pooled adjusted OR of 1.191 (95% CI: 1.023–1.359) (S7 Fig). The effect was statistically significant (z = 13.87, p < 0.001). However, there was substantial heterogeneity among these studies, with Cochran’s Q = 17.10 (p = 0.004), an I² of 70.8%, and a between-study variance (tau²) of 0.0233. We further stratified the analysis of functional outcomes by the risk of bias. In the low risk subgroup, the pooled adjusted OR was 1.153 (95% CI: 0.791–1.514). For the moderate risk subgroup, the pooled adjusted OR was 1.240 (95% CI: 1.020–1.460). In the high risk subgroup, adjusted OR was 1.360 (95% CI: 1.160–1.590) (S8 Fig). Analysis based on the follow-up duration could not be done due to limitation of information related to follow-up time for the outcome in the included studies.

## Discussion

Our analysis demonstrated that NT-proBNP levels are significantly associated with both unadjusted and adjusted mortality in ischemic stroke patients. Specifically, higher NT-proBNP levels correlated with increased mortality rates, with unadjusted odds ratios showing a more pronounced effect compared to adjusted ratios. There was also a marked association between elevated NT-proBNP levels and higher risk of cardiovascular mortality, emphasizing the relevance of this biomarker in predicting severe post-stroke outcomes. Our results are in agreement with previous research that have identified NT-proBNP as a strong prognostic marker of cardiovascular conditions and post-stroke outcomes [35,36]. Numerous reports have consistently demonstrated that high NT-proBNP levels are indicative of cardiac stress and damage, which can also be extrapolated to the cerebrovascular events examined in our review [35–37].

The included studies suggest that higher NT-proBNP levels may be linked to poorer functional and neurological outcomes, although these associations were not statistically significant in all analyses. Therefore, NT-proBNP may potentially serve as a marker for the severity of stroke and patient’s prognosis. The link between NT-proBNP levels and haemorrhagic transformation after the stroke were not statistically significant. This implies that while NT-proBNP is indicative of other severe outcomes, it may not be as reliable for predicting these specific complications.

The significant association between NT-proBNP levels and various stroke outcomes can be understood through several physiological mechanisms. NT-proBNP is released in response to ventricular strain, which may occur as a result of acute neurological injury like stroke [38]. The brain-heart axis suggests that stroke-induced neurological impairment can lead to cardiac dysfunction, which in turn is reflected by elevated NT-proBNP levels [39]. Stroke can lead to disruption of the blood-brain barrier, promoting inflammation and neurohormonal activation that might stimulate NT-proBNP release [40]. Higher NT-proBNP levels could thus indicate more severe cerebral injury. Elevated NT-proBNP levels have been linked to poorer outcomes in various cardiac conditions due to their association with underlying cardiovascular diseases, which are also risk factors for worse outcomes in stroke patients.

Our analyses revealed that the association between elevated NT-proBNP levels and mortality is notably stronger compared to its association with functional and neurological outcomes. One potential explanation for this discrepancy is that NT-proBNP primarily reflects systemic hemodynamic stress and underlying cardiovascular pathology rather than direct neurovascular injury. NT-proBNP is released in response to increased cardiac wall stress, and its elevation may be more indicative of systemic factors—such as pre-existing heart failure, atrial fibrillation, or other cardiovascular conditions—that contribute to overall mortality risk. In contrast, functional and neurological outcomes post-stroke are influenced by a broader spectrum of factors, including the extent of cerebral injury, collateral circulation, and post-stroke rehabilitation efforts, which might not be directly captured by NT-proBNP levels. This suggests that while NT-proBNP is a robust predictor of mortality, its utility in predicting long-term functional or neurological recovery may be limited. Future studies could explore combining NT-proBNP with more neuro-specific biomarkers to improve prognostication in these domains.

Understanding the mechanisms by which NT-proBNP levels influence stroke outcomes might open new therapeutic avenues aimed at mitigating neurocardiac effects in stroke survivors.

Our findings support the prognostic value of NT-proBNP in ischemic stroke; however, translating these results into routine clinical practice presents several challenges. First, a universal cutoff for NT-proBNP is unlikely to be applicable given the substantial heterogeneity in patient characteristics—such as age, renal function, and pre-existing cardiac conditions—that can influence baseline NT-proBNP levels. Instead, NT-proBNP should be integrated into multifactorial risk stratification models rather than used as a stand-alone indicator.

Furthermore, evidence suggests that biological variability accounts for approximately 40% of NT-proBNP alterations [41], which further complicates its use in clinical decision-making. This high degree of inherent variability implies that single measurements may not reliably reflect a patient’s true risk. Serial measurements and monitoring trends over time might offer more clinically relevant insights. Clinicians should interpret NT-proBNP levels in the context of other clinical findings and biomarkers, tailoring risk assessments to the individual patient profile.

NT-proBNP levels are known to be influenced by underlying cardiovascular conditions, such as atrial fibrillation and heart failure. To mitigate this potential confounding, many of the included studies adjusted for these factors in their multivariable models. Although the specific covariates varied across studies, several common adjustments were noted. Most studies controlled for demographic factors (e.g., age and sex) and traditional cardiovascular risk factors, including hypertension, diabetes, and hyperlipidemia. In addition, many studies specifically adjusted for a history of cardiovascular conditions (e.g., atrial fibrillation, heart failure) to better isolate the prognostic value of NT-proBNP in the context of ischemic stroke.

Moreover, several investigations further incorporated measures of stroke severity—such as the National Institutes of Health Stroke Scale (NIHSS)—and other acute stroke-related parameters (e.g., time from symptom onset to NT-proBNP measurement) into their adjusted models. Although the list of covariates was not uniform across studies, these adjustments consistently aimed to account for the influence of pre-existing cardiovascular pathology. This variability in adjustment strategies may partially contribute to heterogeneity in the reported effect sizes, and future research would benefit from a more standardized approach to covariate selection.

This review has certain limitations. There was significant heterogeneity in our meta-analyses, which could be due to differences in study populations, NT-proBNP measurement timing, and stroke severity. Variability in how studies utilized and categorized the biomarker of interest was another limitation. Some studies divided levels using categorical thresholds, others employed quintiles or quartiles, while some analyzed it as a continuous variable. This lack of standardized cutoff values poses challenges in translating findings into clinical practice, as it limits the biomarker’s applicability and reliability for routine clinical use.

Another limitation of this study is the potential influence of hemodynamic status on NT-proBNP levels, which was not consistently described or controlled for in most of the included studies. Hemodynamic factors, such as volume status, cardiac output, and blood pressure, are known to significantly impact NT-proBNP levels, potentially confounding the observed associations with stroke outcomes. The absence of systematic reporting and adjustment for these factors limits the ability to generalize the findings across diverse clinical contexts. Despite the overall robustness of our findings, asymmetry observed in funnel plots and significant Egger’s test indicate presence of potential publication bias. This bias may arise because studies reporting non-significant or negative associations between NT-proBNP levels and ischemic stroke outcomes are less likely to be published. Consequently, pooled effect estimates in our analysis could be overestimated, thereby affecting the reliability of conclusions drawn. Although sensitivity analyses and subgroup assessments (e.g., by timing and risk of bias) consistently demonstrated significant associations, the possibility of publication bias suggests that these results should be interpreted with caution. Future studies, including the incorporation of unpublished data and prospective trial registration, are warranted to further clarify and validate these associations.

NT-proBNP could be used as a biomarker for risk stratification in stroke patients. Identifying high-risk patients early could help in tailoring aggressive therapeutic strategies to prevent severe outcomes. Regular monitoring of NT-proBNP levels in stroke patients could provide insights into their recovery trajectory and help in adjusting management plans accordingly.

By predicting functional and neurological outcomes, NT-proBNP levels could guide rehabilitation efforts, focusing resources on patients who are at higher risk of poor recovery.

Future research should aim to establish standardized protocols for NT-proBNP measurement in the acute and subacute phases of stroke and explore its incorporation into existing clinical scoring systems. By doing so, NT-proBNP could contribute to more precise risk stratification and targeted management strategies, ultimately improving the care and outcomes of stroke patients. More long-term longitudinal studies are needed to observe the progression of NT-proBNP levels from acute to chronic phases of stroke recovery and to assess how changes in these levels correlate with patient outcomes over time. Investigating the biological mechanisms through which NT-proBNP influences stroke outcomes can help clarify its role as a biomarker. Such studies could explore the impact of stroke event on neurocardiac interactions, particularly in patients with pre-existing cardiovascular conditions. Future research should also focus on standardizing timing and methods of NT-proBNP measurement after the stroke to reduce heterogeneity across studies, and making findings more comparable. Exploring NT-proBNP in conjunction with other biomarkers could provide a more comprehensive prognostic model to more accurately predict stroke outcomes.

## Conclusion

The review has underscored significant prognostic value of NT-proBNP for various outcomes in ischemic stroke patients, including mortality, cardiovascular mortality, and functional recovery. The consistent association across multiple studies highlights the potential of NT-proBNP as a reliable biomarker for assessing stroke severity and predicting patient outcomes.

## Supporting information

S1 Fig Funnel plot for all-cause mortality. (TIF)

S2 Fig Subgroup analysis based on NT-proBNP measurement at the time of admission for the association between N-terminal pro brain natriuretic peptide levels and mortality amongst ischaemic stroke patients. (JPG)

S3 Fig Subgroup analysis based on NT-proBNP measurement at < 24 hours for the association between N-terminal pro brain natriuretic peptide levels and mortality amongst ischaemic stroke patients. (JPG)

S4 Fig Subgroup analysis based on risk of bias for the association between N-terminal pro brain natriuretic peptide levels and mortality amongst ischaemic stroke patients. (JPG)

S5 Fig Funnel plot for functional outcomes. (TIF)

S6 Fig Subgroup analysis based on study design showing the association between N-terminal pro brain natriuretic peptide levels and functional outcomes amongst ischaemic stroke patients. (TIF)

S7 Fig Subgroup analysis based on NT-proBNP measurement at < 24 hours for the association between N-terminal pro brain natriuretic peptide levels and functional outcomes amongst ischaemic stroke patients. (JPG)

S8 Fig Subgroup analysis based on risk of bias for the association between N-terminal pro brain natriuretic peptide levels and functional outcomes amongst ischaemic stroke patients. (JPG)

S1 File List of excluded studies with reasons. (XLSX)

S2 File Dataset. (XLSX)

S3 File PRISMA checklist. (DOCX)
