# Supplementary figures and images for: Association between N-terminal pro-brain natriuretic peptide levels and outcomes of ischemic stroke: A systematic review and meta-analysis
Source: PLoS One. 2025 Jun 27;20(6):e0322816. doi: 10.1371/journal.pone.0322816 (PMC12204559; doi:10.1371/journal.pone.0322816)

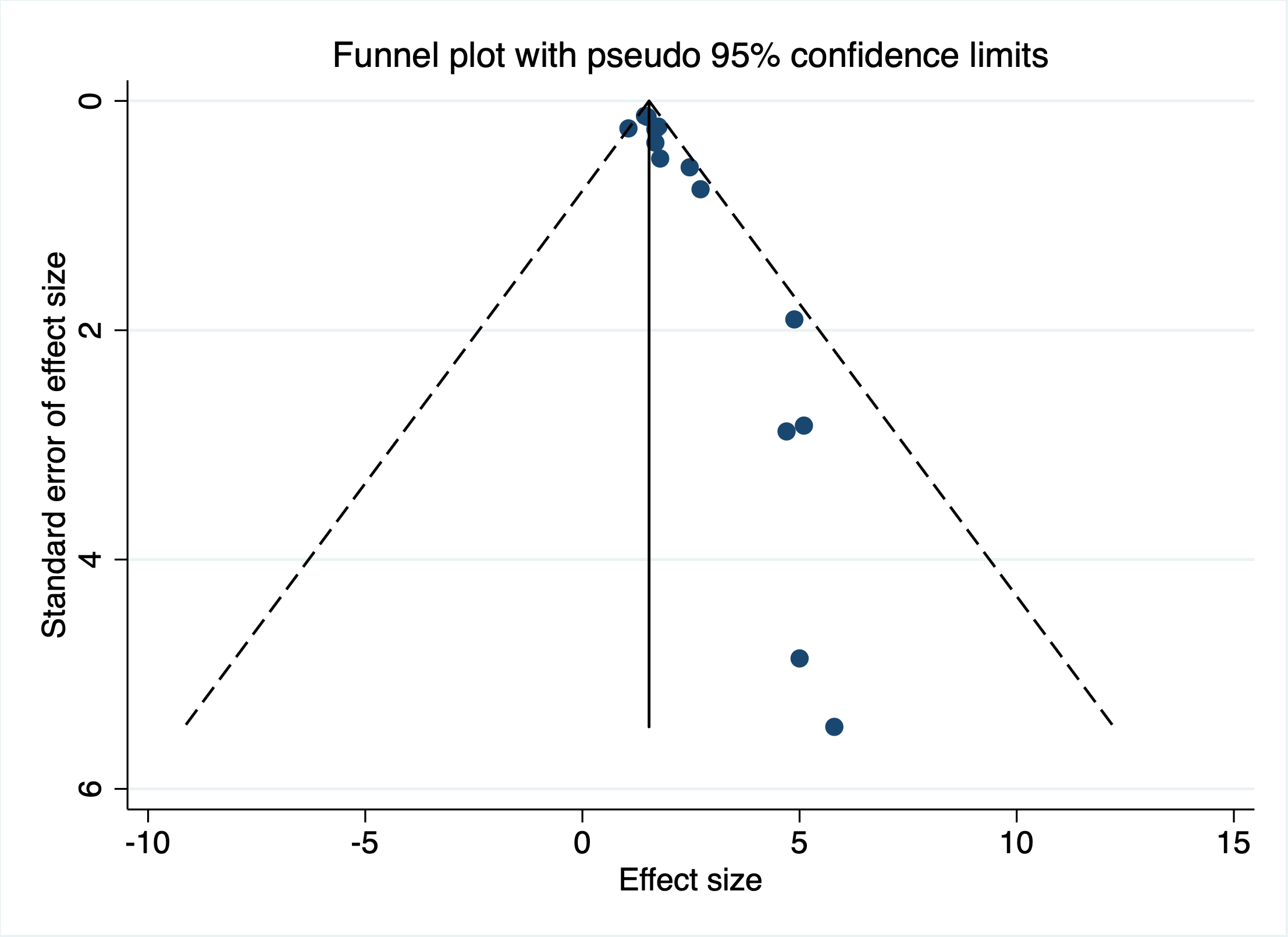

Supplement: S1 Fig — (TIF) [file pone.0322816.s001.tif]

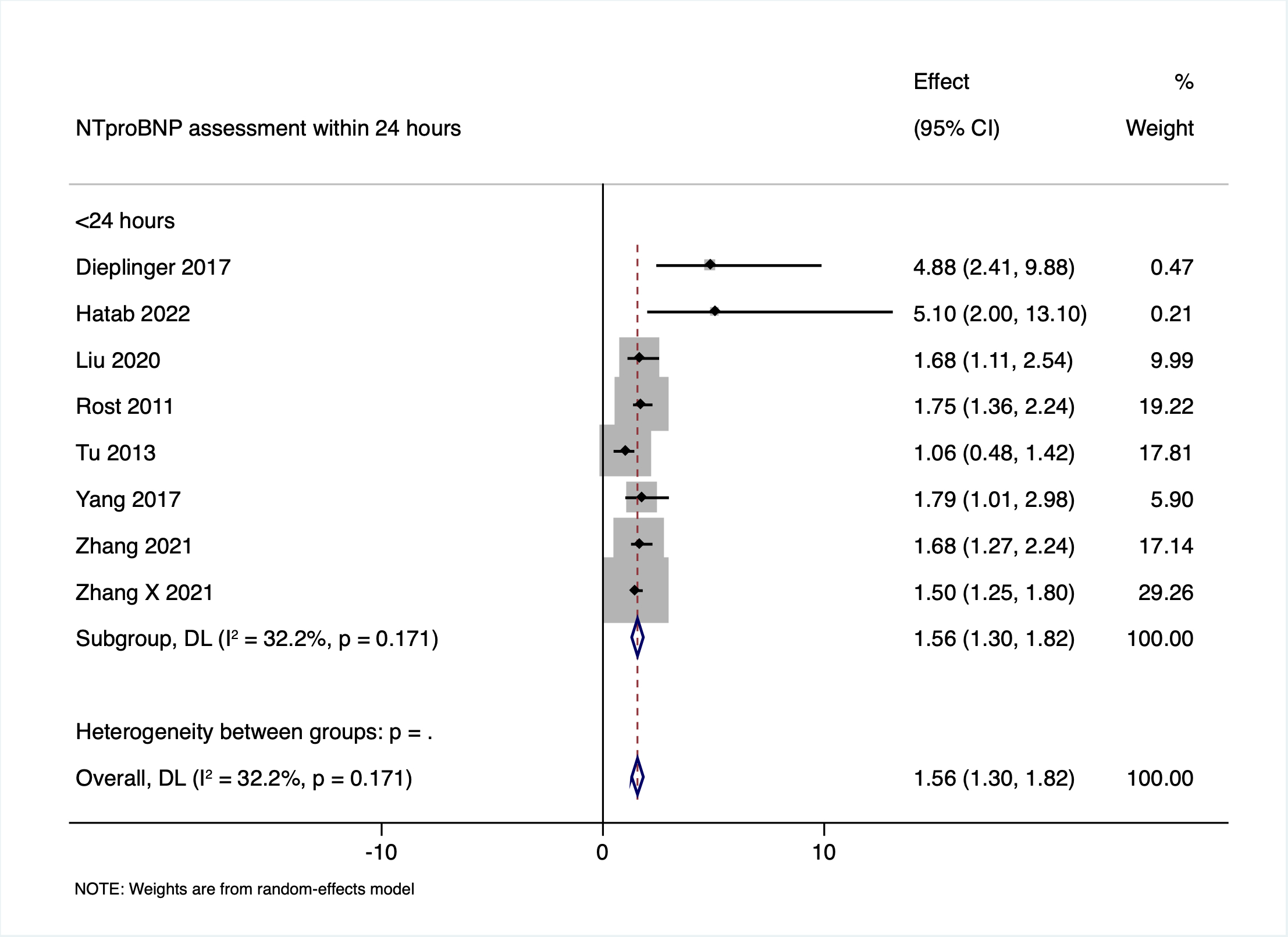

Supplement: S2 Fig — (JPG) [file pone.0322816.s002.jpg]

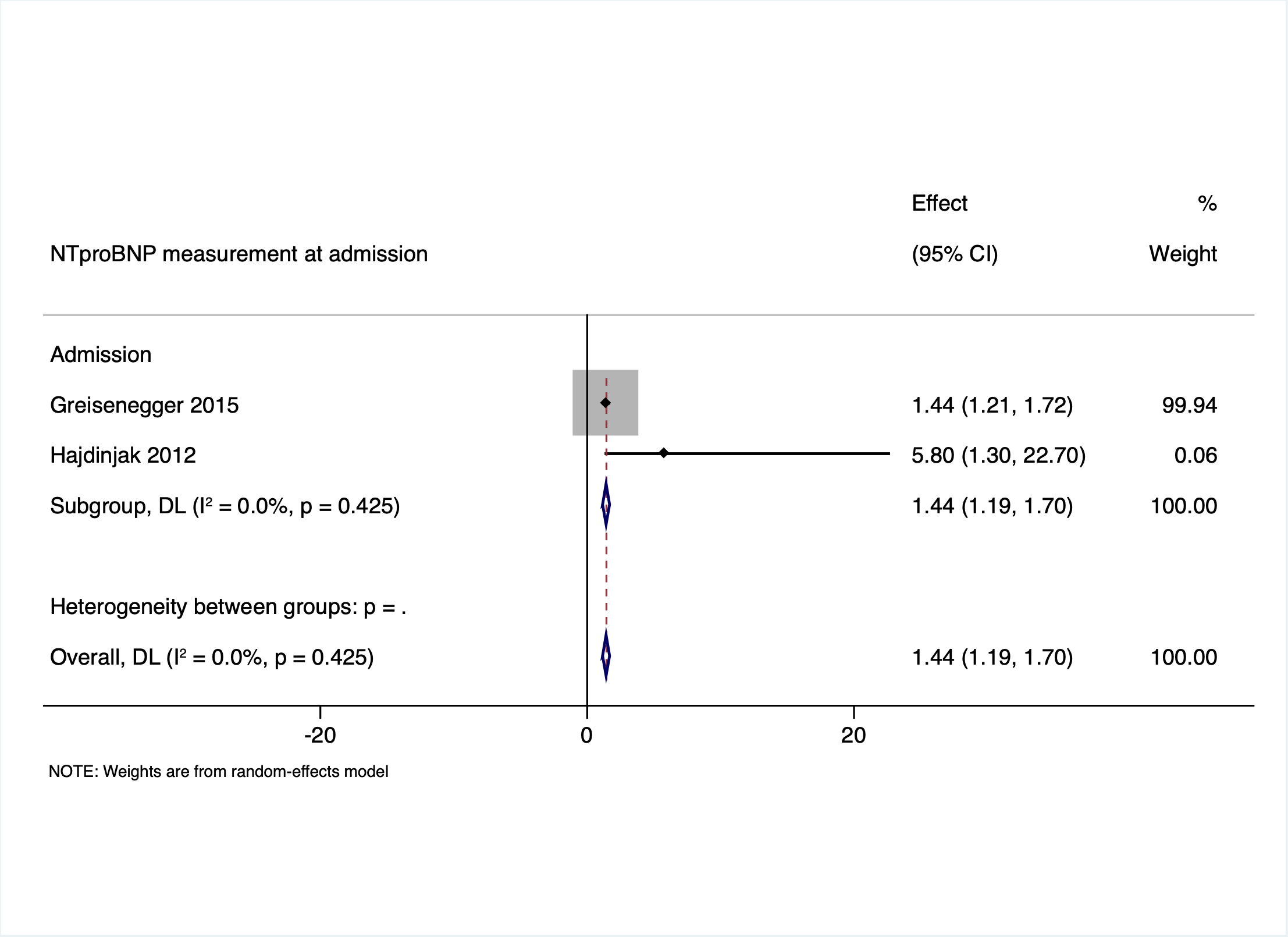

Supplement: S3 Fig — (JPG) [file pone.0322816.s003.jpg]

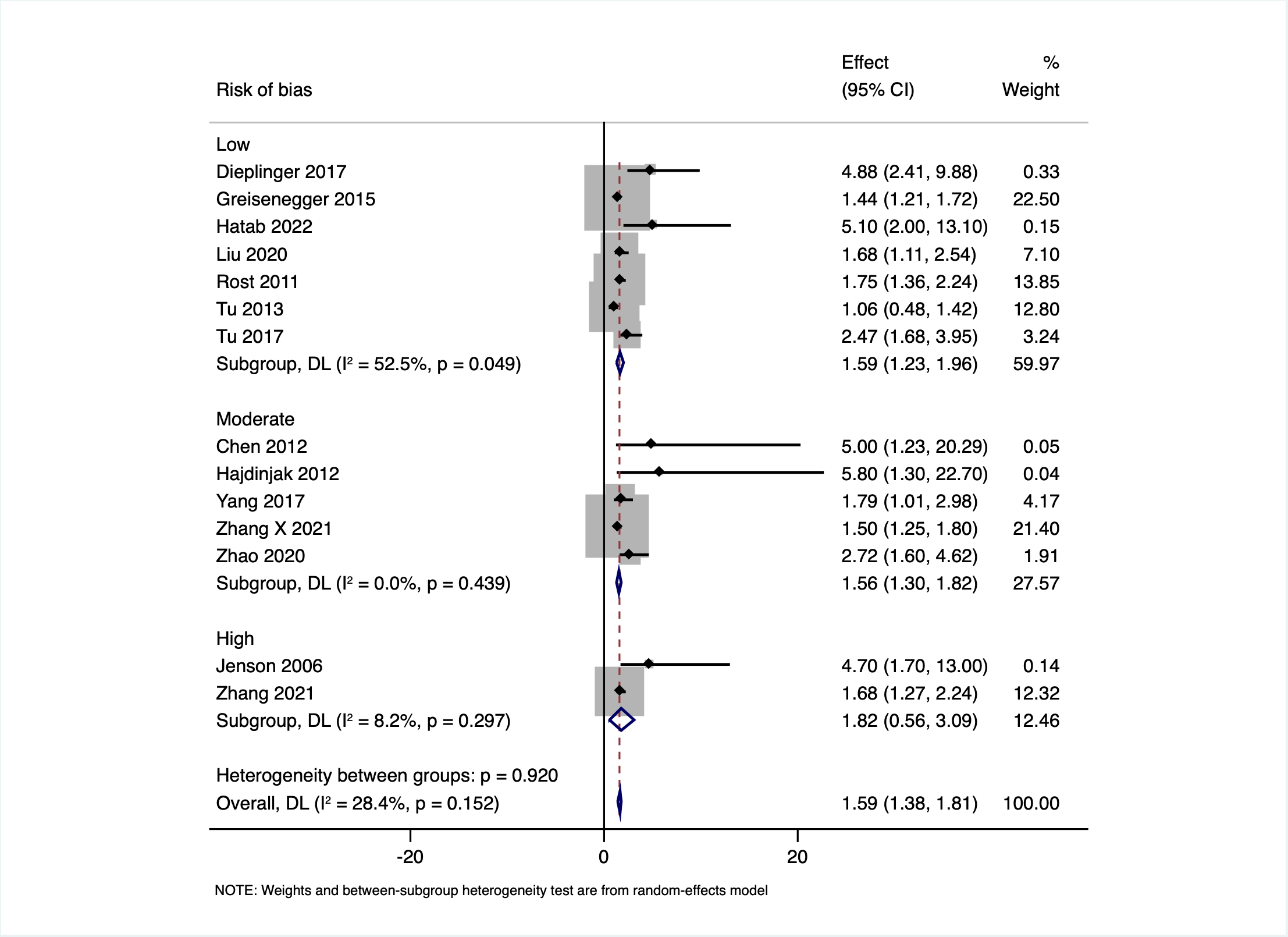

Supplement: S4 Fig — (JPG) [file pone.0322816.s004.jpg]

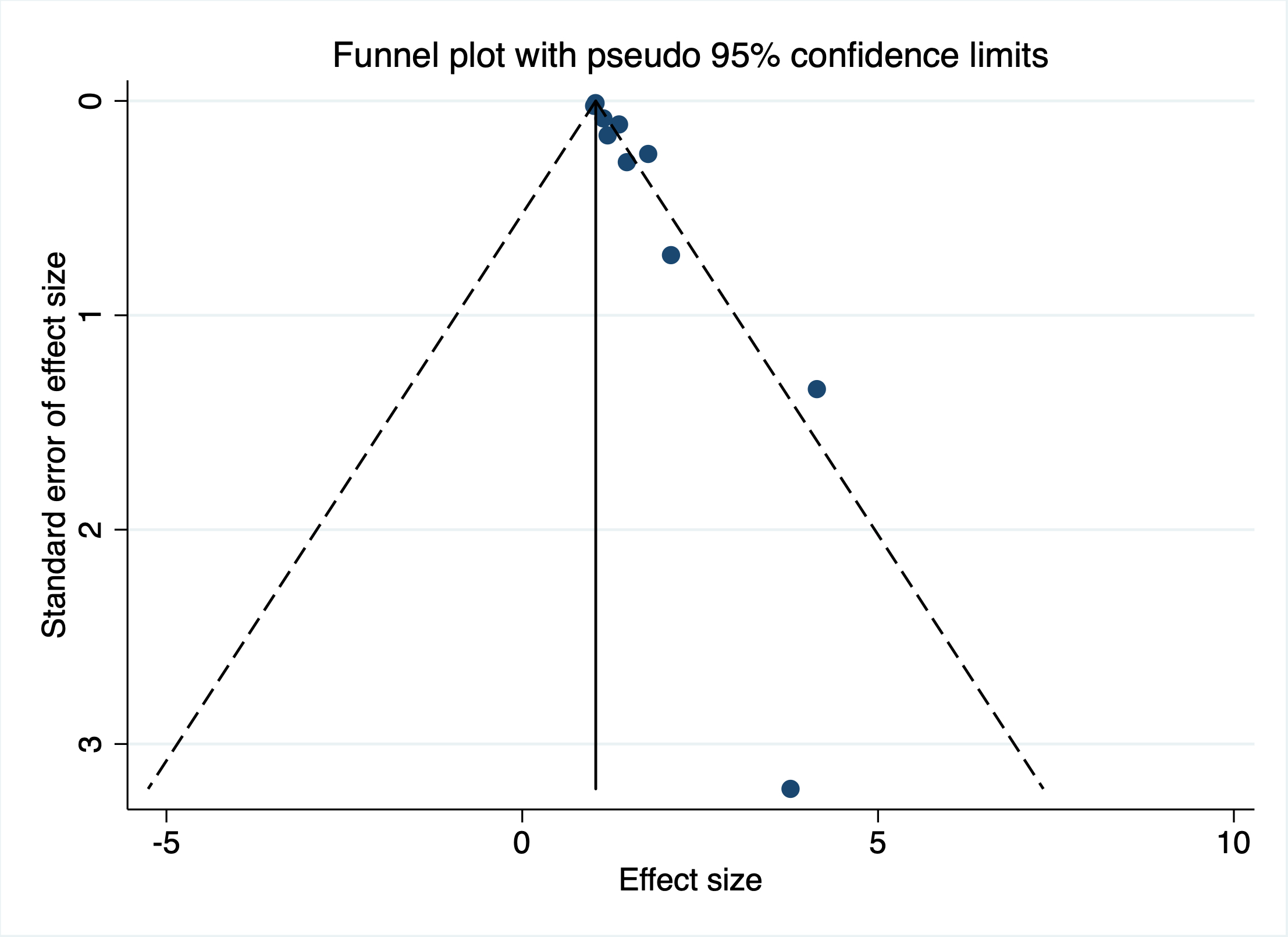

Supplement: S5 Fig — (TIF) [file pone.0322816.s005.tif]

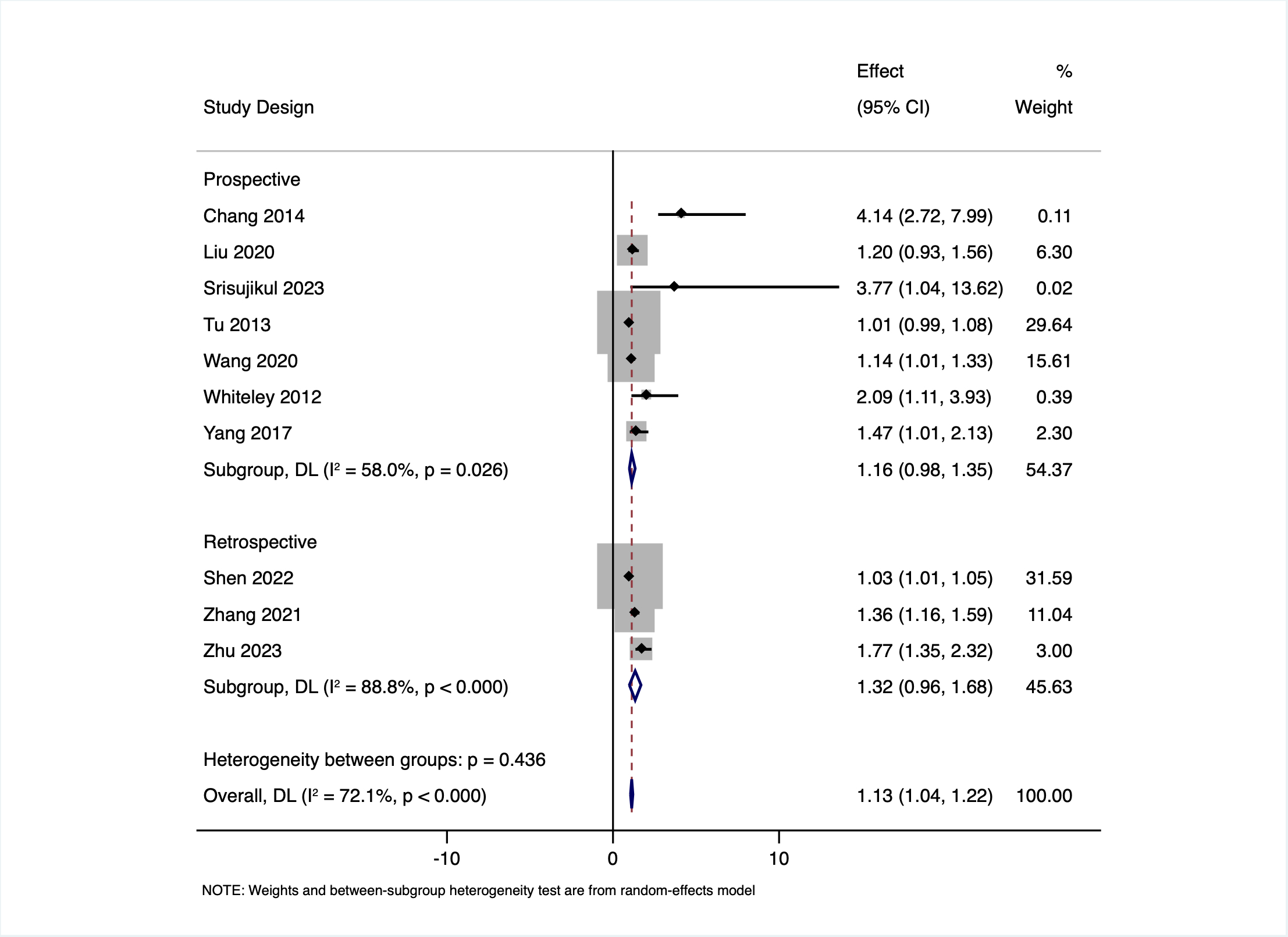

Supplement: S6 Fig — (TIF) [file pone.0322816.s006.tif]

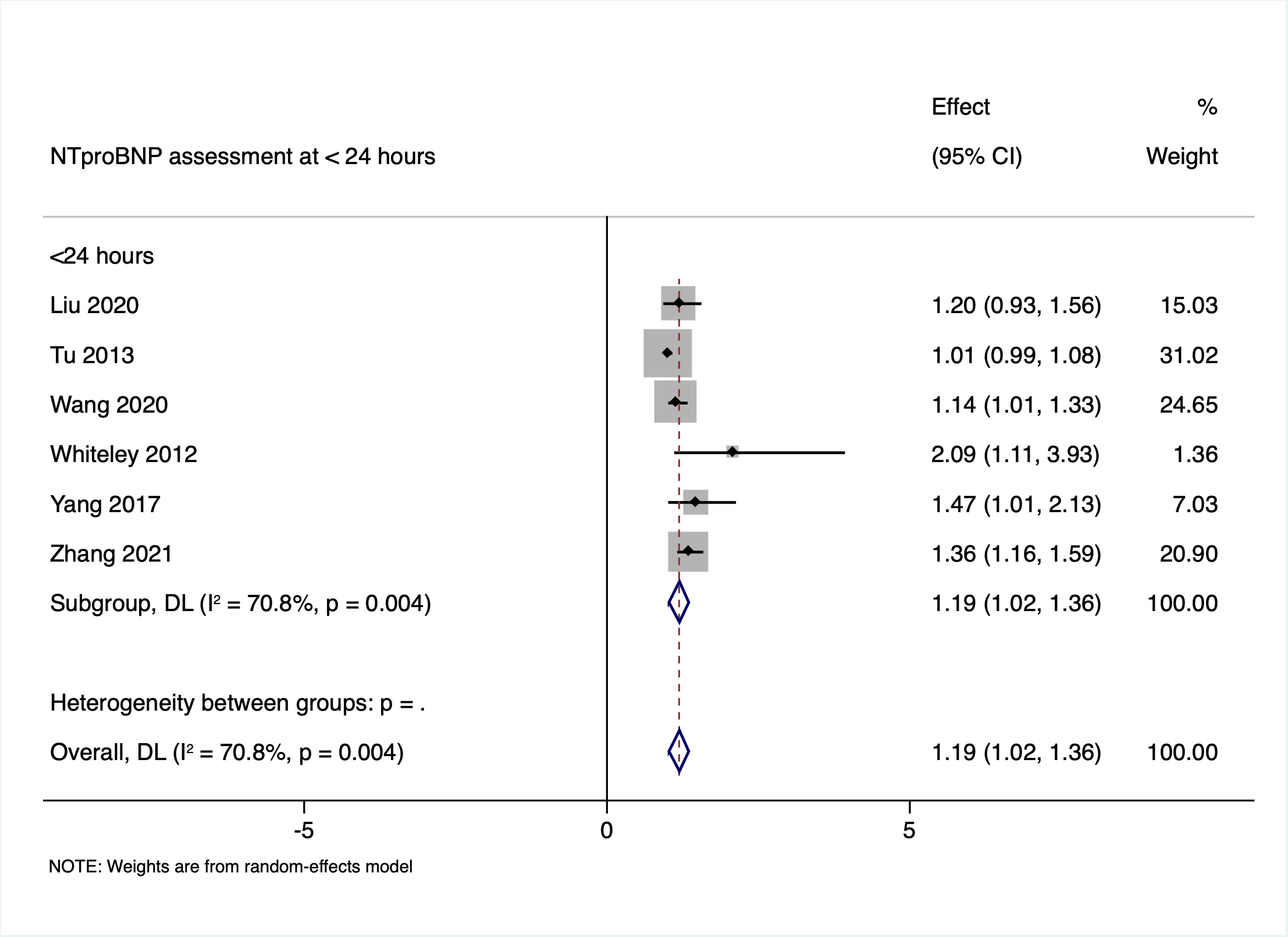

Supplement: S7 Fig — (JPG) [file pone.0322816.s007.jpg]

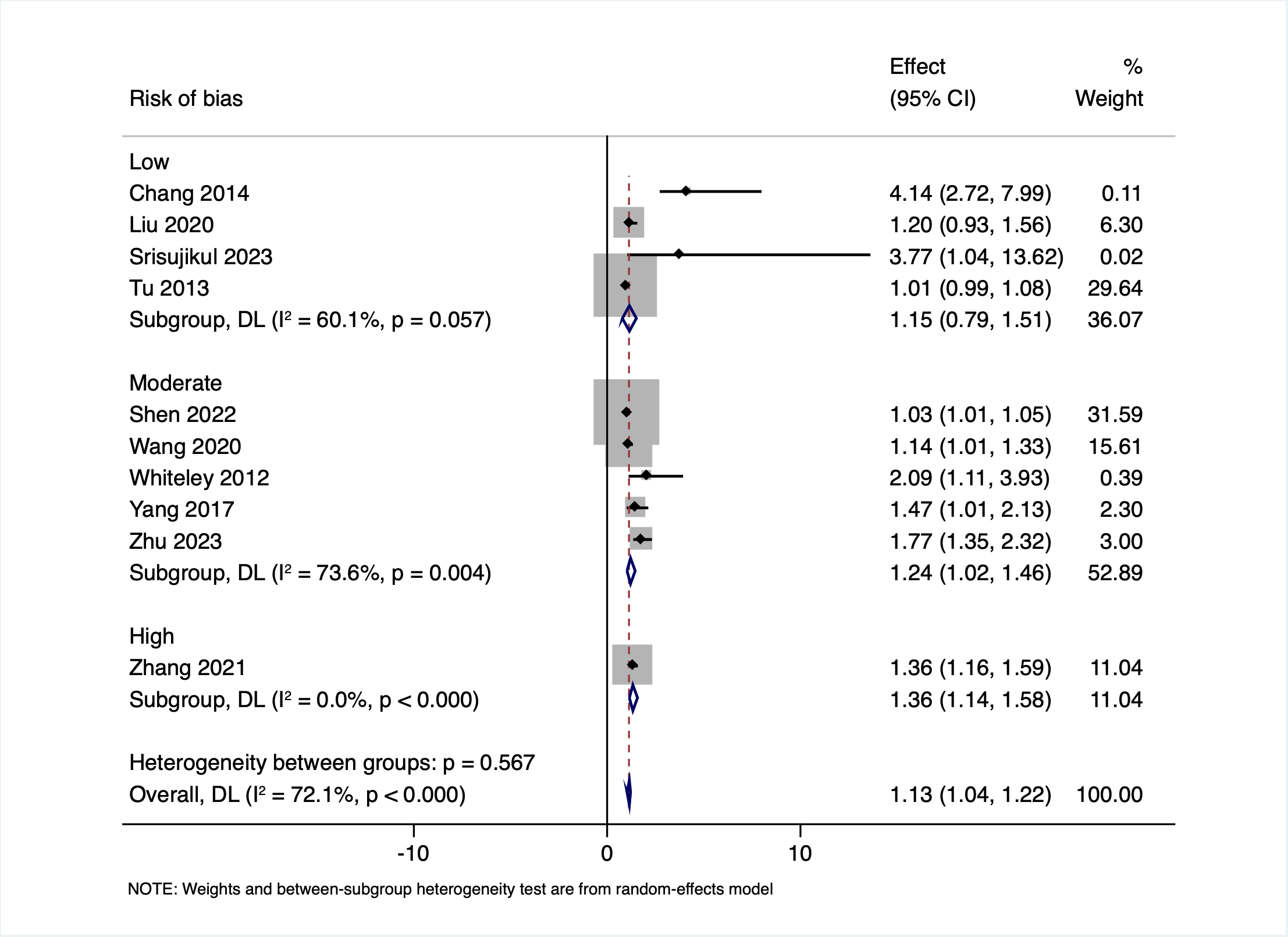

Supplement: S8 Fig — (JPG) [file pone.0322816.s008.jpg]
